# Differential Leukocyte Expression of *IFITM1* and *IFITM3* in Patients with Severe Pandemic Influenza A(H1N1) and COVID-19

**DOI:** 10.1089/jir.2022.0036

**Published:** 2022-08-18

**Authors:** Nora E. Regino-Zamarripa, Gustavo Ramírez-Martínez, Luis Armando Jiménez-Álvarez, Alfredo Cruz-Lagunas, Itzel Alejandra Gómez-García, Sergio Ignacio-Cortés, José Eduardo Márquez-García, Lynette Miroslava Pacheco-Hernández, Jazmín Ariadna Ramírez-Noyola, Rodrigo Barquera, Criselda Mendoza-Milla, Cesar Luna-Rivero, José Guillermo Domínguez-Cherit, Remedios Ramírez-Rangel, Tatiana Sofía Rodríguez-Reyna, Carmen M. Hernández-Cárdenas, José Alberto Choreño-Parra, Gloria León-Ávila, Joaquín Zúñiga

**Affiliations:** ^1^ Laboratory of Immunobiology and Genetics, Instituto Nacional de Enfermedades Respiratorias “Ismael Cosío Villegas,” Mexico City, Mexico.; ^2^ Programa de Doctorado en Ciencias Quimicobiológicas, Sección de Estudios de Posgrado e Investigación, Escuela Nacional de Ciencias Biológicas, Instituto Politécnico Nacional, Prolongación de Carpio and Plan de Ayala s/n, Mexico City, Mexico.; ^3^ Tecnologico de Monterrey, Escuela de Medicina y Ciencias de la Salud, Mexico City, Mexico.; ^4^ Programa de Maestría en Ciencias de la Salud, Sección de Estudios de Posgrado e Investigación, Escuela Superior de Medicina, Instituto Politécnico Nacional, Salvador Díaz Mirón and Plan de San Luis, Mexico City, Mexico.; ^5^ Department of Archaeogenetics, Max Planck Institute for Science of Human History, Jena, Germany.; ^6^ Deparment of Pathology, Instituto Nacional de Enfermedades Respiratorias “Ismael Cosío Villegas,” Mexico City, Mexico.; ^7^ Critical Care Unit, Instituto Nacional de Ciencias Médicas y Nutrición “Salvador Zubirán, Mexico City, Mexico.; ^8^ Facultad de Ciencias, Universidad Nacional Autónoma de México, Investigación Científica, Mexico City, Mexico.; ^9^ Department of Immunology and Rheumatology, Instituto Nacional de Ciencias Médicas y Nutrición “Salvador Zubirán, Mexico City, Mexico.; ^10^ Respiratory Critical Care Unit, Instituto Nacional de Enfermedades Respiratorias “Ismael Cosío Villegas,” Mexico City, Mexico.; ^11^ Zoology Deparment, Escuela Nacional de Ciencias Biológicas, Instituto Politécnico Nacional, Prolongación de Carpio and Plan de Ayala s/n, Mexico City, Mexico.

**Keywords:** pandemic influenza, influenza A(H1N1), IFITM1, IFITM3, pneumonia

## Abstract

Interferon-induced transmembrane (IFITM) proteins mediate protection against enveloped viruses by blocking membrane fusion at endosomes. IFITM1 and IFITM3 are crucial for protection against influenza, and various single nucleotide polymorphisms altering their function have been linked to disease susceptibility. However, bulk *IFITM1* and *IFITM3* mRNA expression dynamics and their correlation with clinical outcomes have not been extensively addressed in patients with respiratory infections. In this study, we evaluated the expression of *IFITM1* and *IFITM3* in peripheral leukocytes from healthy controls and individuals with severe pandemic influenza A(H1N1) or coronavirus disease 2019 (COVID-19). Comparisons between participants grouped according to their clinical characteristics, underlying disease, and outcomes showed that the downregulation of *IFITM1* was a distinctive characteristic of severe pandemic influenza A(H1N1) that correlated with outcomes, including mortality. Conversely, increased *IFITM3* expression was a common feature of severe pandemic influenza A(H1N1) and COVID-19. Using a high-dose murine model of infection, we confirmed not only the downregulation of *IFITM1* but also of *IFITM3* in the lungs of mice with severe influenza, as opposed to humans. Analyses in the comparative cohort also indicate the possible participation of IFITM3 in COVID-19. Our results add to the evidence supporting a protective function of IFITM proteins against viral respiratory infections in humans.

## Introduction

Influenza is still a leading cause of respiratory morbidity and mortality worldwide. The disease burden derives from seasonal winter outbreaks and unpredictable sporadic pandemics of variable magnitude, the last occurring in 2009 after the emergence of a novel A(H1N1) influenza virus (Centers for Disease Control and Prevention 2009; Novel Swine-Origin Influenza and others 2009; Perez-Padilla and others 2009). Notably, pandemic influenza viruses have an increased capacity to suppress innate antiviral defenses, thus affecting young adults and inducing severe manifestations with a higher frequency than seasonal viruses. For instance, the influenza A(H1N1) pdm09 virus suppresses type I interferon (IFN) responses of dendritic cells and macrophages (Osterlund and others 2010; Ramírez-Martínez and others 2013), reducing the activation of several IFN-stimulated antiviral mechanisms.

Interferon-induced transmembrane (IFITM) proteins are among the host factors that restrict viral infection by interfering with cell entry at endosomes (Brass and others 2009). As such, members of the IFITM family mediate early cellular resistance against a broad list of enveloped viral pathogens, including influenza virus (Brass and others 2009; Everitt and others 2012; Jia and others 2012; Smith and others 2013; Lanz and others 2015; Yu and others 2015; Blyth and others 2016; Meischel and others 2021; Rohaim and others 2021), West Nile virus, dengue virus (Brass and others 2009), rabies virus (Wang and others 2020), human immunodeficiency virus (Jia and others 2012; Chutiwitoonchai and others 2013), Zika virus, vesicular stomatitis virus (Alber and Staeheli 1996; Chesarino and others 2017), chikungunya virus, Mayaro virus (Franz and others 2021), hepatitis C virus (HCV)(Zhu and Liu 2003), severe acute respiratory syndrome coronavirus (SARS-CoV), Marburg virus, and Ebola virus (Huang and others 2011; Chesarino and others 2017).

Although the protective mechanisms of IFITM proteins are not entirely understood, *in vitro* assays and animal studies show that IFITM1 might interfere with the fusion of viral and early endosome/lysosome membranes, whereas IFITM3 blocks membrane fusion in late endosomes (Brass and others 2009; Feeley and others 2011; Jia and others 2012; Li and others 2013; Chesarino and others 2017). Interestingly, influenza viruses can directly alter the regulation of IFITM protein expression to avoid their functions (Wang and others 2018). Moreover, recent clinical investigations in humans have linked increased susceptibility to influenza with specific single nucleotide polymorphisms (SNPs) in genes coding IFITM1 and IFITM3 (Everitt and others 2012; Zhang and others 2013; Allen and others 2017; Kim and others 2020, 2021).

Certain alleles carrying SNPs modify IFITM protein structure and localization to endosomes, limiting their antiviral activities or affecting IFITM-dependent accumulation of immune cells in the airways (Allen and others 2017; Kim and others 2020; Franz and others 2021).

Despite these advances, the dynamics of IFITM expression and their relationship with disease severity have not been extensively addressed in humans with influenza and other respiratory infections. Also, little information from cohorts of non-Caucasian populations exists in the literature. Hence, in this study, we evaluated the profile of *IFITM1* and *IFITM3* expression in peripheral leukocytes from Latin American patients with severe pandemic influenza A(H1N1). Using comparative groups of healthy controls (HC) and individuals with coronavirus disease 2019 (COVID-19), we found that low *IFITM1* and increased *IFITM3* leukocyte expression are distinctive features of pandemic influenza A(H1N1).

Importantly, *IFITM1*, but not *IFITM3*, showed a modest predictive value to identify patients with severe disease at mortality risk. In a high-dose mouse model of pandemic influenza A(H1N1), we observed the downregulation of *IFITM1* in the infected lungs. Meanwhile, *IFITM3* was also suppressed in the pulmonary tissue of mice as opposed to leukocytes from patients with severe pandemic influenza A(H1N1). Analyses in the comparative COVID-19 cohort also suggest the possible participation of IFITM1 and IFITM3 in antiviral defenses against SARS-CoV-2. Our results add to evidence of the importance of IFITM proteins in protective immunity against viral respiratory infections in humans.

## Methods

### Human samples

We recruited 31 consecutive individuals with severe pandemic influenza A(H1N1) from a previously reported cohort (Choreño-Parra and others 2021c, 2021d). Patients attending with acute respiratory distress syndrome (ARDS), who required intensive care unit admission and mechanical ventilation (MV), were eligible for this study if they provided adequate samples for analysis and met the inclusion criteria described before (Choreño-Parra and others 2021c, 2021d). Data and specimens from a comparative cohort of age- and sex-matched HC (*n* = 8) and patients with severe COVID-19 (*n* = 16) were also included in the following analyses. All the enrolled participants reported to be of Mexican admixed race.

Clinical information from study participants was retrieved by direct clinical interview, physical examination, and revision of their medical records. Relevant data for this study included age, gender, anthropometrics, comorbidities, symptoms, triage vital signs, and admission Sequential Organ Failure Assessment (SOFA)/Acute Physiology and Chronic Health Evaluation II (APACHE II) scores. White blood cell counts, liver, and kidney function parameters, procalcitonin (PCT), blood gases, tissue injury markers, and other laboratory test results obtained within 24 h of admission were also retrieved. Patients were followed during their hospitalization, and the incidence of specific complications, requirement of antibiotics, corticosteroids, antivirals, specific intensive care interventions, and outcomes were also registered. Blood samples were obtained from all participants during enrollment and used for peripheral blood mononuclear cell (PBMC) isolation by density gradient as before (Choreño-Parra and others 2021b, 2021c, 2021d).

### IFITM expression in humans

Total ribonucleic acid (RNA) was isolated from PBMCs using the RNeasy Mini kit (Qiagen, Hilden, Germany) and DNase I treated. Total RNA was reverse transcribed using the Revert Aid H minus first-strand cDNA Kit (ThermoFisher Scientific, Waltham, MA) as recommended by the manufacturer. cDNA was then amplified using validated TaqMan assays (Applied Biosystems, Foster City, CA); *IFITM1* assay number: Hs00705137_s1 and *IFITM3* assay number: Hs00829485_sH. Glyceraldehyde 3-phosphate dehydrogenase (*GAPDH*) assay number: Hs02786624_g1. Quantitative real-time-polymerase chain reaction (qRT-PCR) was performed on a Quant Studio 12K Flex (Applied Biosystems). Triplicate cycle threshold *Ct* values were analyzed using the comparative double delta *Ct* method and then presented as mRNA expression relative quantification (RQ) units relative to *GAPDH*.

### Influenza infection in mice

Female wild-type C57BL/6 (B6) mice were bred at the animal facility of the Instituto Nacional de Enfermedades Respiratorias “Ismael Cosío Villegas” (INER) and used between the ages of 6–8 weeks (16–18g) following the Institutional Animal Care and Use Committee guidelines, under the protocol B04-15. Experimental mice were infected intranasally with 1 × 10^6^ plaque-forming units of an influenza A(H1N1) pdm09 virus clinical isolate under isoflurane anesthesia. Weight was measured daily to monitor disease severity. Mice were euthanized with isoflurane upon 30% loss of initial body weight or deemed moribund based on clinical signs. After 7 days postinfection, bronchoalveolar lavage (BAL) was obtained by flushing the airways with 500 μL of saline solution, followed by centrifugation, cell and supernatant collection, and cryopreservation at −20°C.

### IFITM expression in mice

Lung tissues were mechanically homogenized and total lung RNA purified with RNeasy Kit (Qiagen), adding a DNase I treatment step. cDNA was synthesized using the Maxima H Minus First Strand cDNA Synthesis Kit (ThermoFisher Scientific). The RT-PCR reaction was set up in duplicate with the Maxima Probe/ROX qPCR Master Mix (ThermoFisher Scientific) using commercial TaqMan primers for *IFITM1* (Mm00850040_g1), *IFITM3* (Mm00847057_s1), and the endogenous control *GAPDH* (Mm99999915_g1) and run in a Step One Real-Time PCR System (ThermoFisher Scientific) following the manufacturer's protocol. The double delta *Ct* method was used to quantify the relative mRNA expression of target genes.

### Cytokine levels in mouse lungs

BAL samples were analyzed for determining concentrations of interleukin (IL)-2, IL-4, IL-5, IL-10, IL-12(p70), IFN-γ, tumor necrosis factor (TNF), and granulocyte-macrophage colony-stimulating factor (GM-CSF) using a magnetic bead-based Luminex multiplex cytokine assay (Bio-Rad Corp., Hercules, CA). Briefly, the BAL samples were mixed with antibody-linked polystyrene beads in a 96-well magnetic bottom plate and incubated at RT for 30 min on an orbital shaker set at 500 rpm. After washing, the plates were incubated with a biotinylated detection antibody for 30 min at RT. The plates were then washed twice and resuspended in a streptavidin-PE solution. After a 15-min incubation, 2 additional washes were performed, and the samples were resuspended in a reading buffer. Each sample was measured in parallel with an 8-point serial dilution standard curve and buffer-only controls. The plates were read using a Luminex Bio-plex 200 system (Bio-Rad Corp.).

### Histology

Mouse lungs were harvested at given time points, inflated with 10% neutral buffered formalin, and embedded in paraffin. Five-micrometer mouse lung sections were stained with hematoxylin and eosin and processed for light microscopy.

Tissue sections were processed for immunohistochemistry (IHQ) and incubated overnight at RT with optimal dilutions of the following antibodies: anti-human/mouse IFITM1 (1:200; Biovision, UK) and anti-human/mouse IFITM3 (:200; Biovision), as previously described (Choreño-Parra and others 2021a). Briefly, human- and mice-fixed tissue sections were deparaffinized, rehydrated, and then blocked with 3% H_2_O_2_ in methanol for 30 min, followed by antigen retrieval performed with citrate buffer 10 mM, pH 6.0, for 5 min in the microwave. Slides were incubated with antibodies and then with a secondary biotinylated anti-immunoglobulin antibody followed by horseradish peroxidase-conjugated streptavidin (BioGenex, San Ramon, CA).

The primary antibody was replaced by nonimmune serum for negative controls, and attached antibodies revealed with 3-amino-9-ethyl-carbazole (BioGenex) as a substrate in acetate buffer containing 0.05% H_2_O_2_. The tissue sections were counterstained with hematoxylin. The data obtained by IHQ were interpreted as staining intensity, according to direct observation by an evaluator who determined the fraction of positive cells for each protein in each tissue (low, <25%; medium, 25%–75%; and high >75%).

### Study approval

This study was reviewed and approved by the Institutional Review Boards of participating institutions under the approval numbers INER/B04-15/B28-16/B09-20 and INCMNSZ/3349. All participants or their legal guardians provided written consent to participate in the study. Serum samples were managed according to the national law NOM-012-SSA3-2012, which establishes the criteria for executing clinical investigations in humans.

### Statistical analysis

Descriptive statistics were used to characterize the study cohorts. Frequencies and proportions were calculated for categorical data. Medians, interquartile ranges, and 95% confidence intervals were used for continuous variables. Patients were grouped according to their underlying disease, outcome (survival versus death), or specific complications during the follow-up period. Comparisons between human and animal groups were made using a Fisher's exact test, unpaired Mann–Whitney *U* test, or Kruskal–Wallis with *post hoc* Dunn's test, as appropriate.

Multiple linear regression analyses using Spearman rank correlation coefficients were used to determine correlations between continuous clinical variables, cytokine levels, and relative expression values of *IFITM1* and *IFITM3* in humans and mice. The diagnostic accuracy of *IFITM1* and *IFITM3* expression to differentiate between participant groups was further evaluated by area under the receiver operating characteristic (ROC) curve (AUC) analyses. In addition, Kaplan–Meier curves were constructed to look for differences in survival according to levels of *IFITM1* and *IFITM3* expression dichotomized by the AUC threshold with the highest diagnostic accuracy estimated using the Youden index.

All analyses were conducted using GraphPad Prism 8 (La Jolla, CA). Specific analytical tests are also described in the figure legends. Values of *P* ≤ 0.05 were considered significant.

## Results

### Participant characteristics

A total of 31 severe pandemic influenza A(H1N1) patients from a previous study had PBMC samples available for this analysis. Their clinical and demographic characteristics recapitulated well the data from the original cohort (Choreño-Parra and others 2021c, 2021d). As such, enrolled patients were preferentially males (∼71%), with a median age of 49 years. Their main comorbidities included obesity (64%), tobacco usage (48%), diabetes (32%), alcoholism (32%), and exposure to biomass smoke (12%). Frequent symptoms reported by patients were fever (93%), dyspnea (87%), myalgia (74%), arthralgia (70%), headache (54%), rhinorrhea (54%), and productive cough (45%).

The clinical data from this cohort are summarized in Table 1. Laboratory parameters measured on admission were characterized by marked lymphopenia, hypoxemia, hypercapnia, elevated lactate dehydrogenase and creatine phosphokinase levels, and high SOFA and APACHE II scores (Table 2). All patients required MV and received oseltamivir and antibiotics during their hospitalization, whereas only 22% were treated with corticosteroids. The most frequent in-hospital complications of influenza were late secondary infections and acute kidney injury (AKIN; Table 1).

**Table 1.** Clinical Characteristics of Patients with Severe Influenza A(H1N1)

Characteristics	*N* = 31
Age (years), median (range)	49 (26–79)
Males	22 (70.96)
BMI	31.4 (28.1–38.2)
Comorbidities	
Smoking	15 (48.38)
Alcoholism	10 (32.25)
Biomass exposure	4 (12.9)
Obesity	20 (64.51)
Diabetes	10 (32.25)
SAH	9 (29.03)
COPD	1 (3.22)
Symptoms at onset	
Fever	29 (93.54)
Myalgia	23 (74.19)
Arthralgia	22 (70.96)
Headache	17 (54.83)
Dyspnea	27 (87.09)
Rhinorrhea	17 (54.83)
Sore throat	10 (32.25)
Thoracic pain	6 (19.35)
Dry cough	13 (41.93)
Productive cough	14 (45.16)
Fatigue	17 (54.83)
Diarrhea	2 (6.45)
Nausea	2 (6.45)
Vomit	1 (3.22)
Duration of symptoms (days), median (range)	7 (7–8)
Triage vital signs	
Body temperature (^o^C)	37.0 (36.6–37.3)
Respiratory rate (bpm)	25 (19–30)
Hearth rate (bpm)	100 (88–109)
MAP (mmHg)	91 (77.6–98)
SO_2_%	80 (70–90)
Stay in hospital (days)	21 (13–32)
Complications	
Acute kidney injury	15 (48.38)
Secondary co-infection	22 (70.96)
Acute myocardial infarction	3 (9.67)
Deep vein thrombosis	2 (6.45)
Stroke	0 (0)
Medical treatment	
Oseltamivir	31 (100)
Antibiotic therapy	31 (100)
No. of antibiotics/patient	5 (3–7)
Corticosteroids	7 (22.58)
Intensive support	
MV	31 (100)
Prone position	16 (51.61)
ECMO	3 (9.67)
RRT	6 (19.35)
Mortality	7 (22.58)

Data are displayed as *n* (%) or median (IQR). *N* is the total number of patients with available data.

BMI, body mass index; bpm, breaths/beats per minute; COPD, chronic obstructive pulmonary disease; ECMO, extracorporeal membrane oxygenation; ICU, intensive care unit; IQR, interquartile range; MAP, mean arterial pressure; MV, mechanical ventilation; PTB, pulmonary tuberculosis; RRT, renal replacement therapy; SAH, systemic arterial hypertension; SO_2_%, oxygen saturation of blood.

**Table 2.** Laboratory Parameters of Severe Influenza A(H1N1) Patients

Parameters	*N* = 31
Blood counts	
WBC (10^9^/L)	6.9 (5.6–10.6)
Neutrophils (10^9^/L)	5.5 (4.5–8.7)
Lymphocytes (10^9^/L)	0.8 (0.5–0.9)
NLR	9.4 (6.0–12.6)
Hgb (g/dL)	15.1 (13.4–17.7)
Platelets (10^9^/L)	157 (124–226)
Metabolic parameters	
Glucose (mg/dL)	135 (112–251)
Na (mmol/L)	136.5 (134–138.5)
K (mmol/L)	4.4 (3.9–4.7)
Ca (mg/dL)	8 (7.8–8.3)
Renal function	
Cr (mg/dL)	1.08 (0.94–1.52)
BUN (mg/dL)	17 (12–29.78)
Liver function	
Total bilirubin (mg/dL)	0.62 (0.43–0.88)
AST (U/L)	67 (53.9–91.2)
ALT (U/L)	41 (29.2–57)
Tissue injury markers	
LDH (U/L)	578 (498–734.7)
ALP (U/L)	109 (93.5–140)
CPK (U/L)	257.4 (130–729.6)
PCT (ng/mL)	0.83 (0.25–2.11)
Respiratory parameters	
pH	7.43 (7.37–7.47)
PCO_2_ (mmHg)	32.1 (25.4–37.4)
PaO_2_ (mmHg)	51 (37.9–59.8)
Lactate (mmol/L)	1.3 (0.9–2)
HCO_3_^-^ (mEq/L)	19.9 (17.3–23.7)
PaO_2_/FiO_2_ (mmHg)	114 (69.8–180)
Severity of illness	
SOFA	6 (5–8)
APACHE II	11 (7–14)

Data are displayed as *n* (%) or median (IQR). *N* is the total number of patients with available data.

ALP, alkaline phosphatase; ALT, alanine aminotransferase; APACHE-II, Acute Physiology and Chronic Health Evaluation II; AST, aspartate aminotransferase; BUN, blood ureic nitrogen; CPK, creatine phosphokinase; Cr, creatinine; FiO_2_, fraction of inspired oxygen; HCO_3_, bicarbonate; Hgb, hemoglobin; K, potassium; LDH, lactate dehydrogenase; Na, sodium; NLR, neutrophil/lymphocyte ration; PaO_2_, partial pressure of oxygen in arterial blood; PCO_2_, partial pressure of carbon dioxide in the blood; PCT, procalcitonin; SOFA, Sequential Organ Failure Assessment; WBC, white blood cell.

The comparative severe COVID-19 group included 16 patients, from which ∼63% were male, with a median age of 42 years. Details about their main comorbidities, symptoms, triage vital signs, laboratory parameters, complications, and treatment are provided in Supplementary Tables S1 and S2. Overall, the 2 populations of severe pandemic influenza A(H1N1) and COVID-19 had similar characteristics, with specific differences that have been extensively commented on before (Choreño-Parra and others 2021c, 2021d) and do not impact the analyses described later. Importantly, therapeutic interventions did not affect the determinations below because clinical specimens were obtained early during hospital admission.

### IFITM1 and IFITM3 in patients with severe pandemic influenza A(H1N1)

**FIG. 1.**
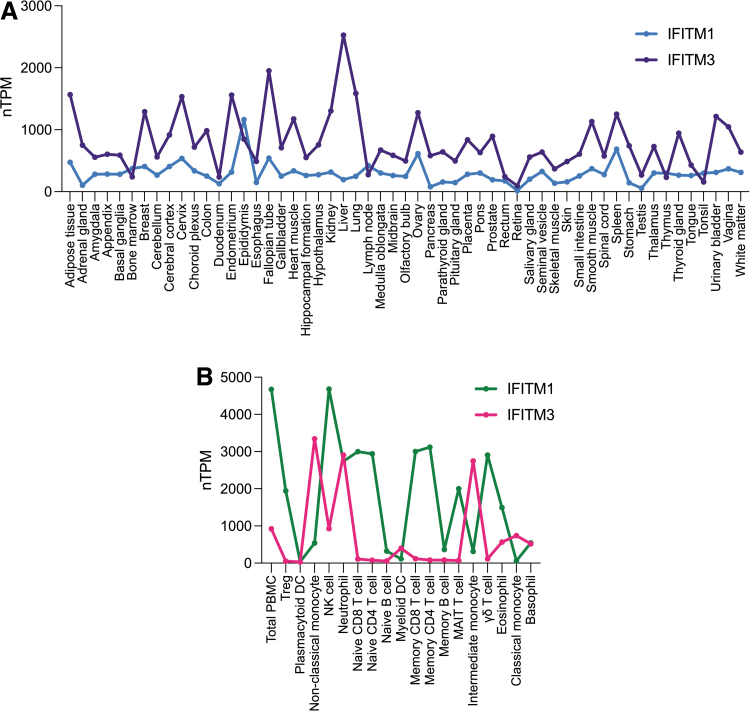
*IFITM1* and *IFITM3* expression in healthy blood cell subsets. **(A)** Expression patterns of *IFITM1* and *IFITM3* in healthy human tissues. **(B)** *IFITM1* and *IFITM3* expression in normal blood cells. mRNA expression in terms of consensus nTPM values of *IFITM1* (ENSG00000185885) and *IFITM3* (ENSG00000142089) was retrieved from the HPA database (www.proteinatlas.org), which includes transcriptomic data of 3 sequencing projects (HPA, GTEx, and FANTOM5) generated by bulk RNA-Seq and single-cell RNAseq data from the Single Cell Expression Atlas, the Human Cell Atlas, the Gene Expression Omnibus, the Allen Brain Map, and the European Genome-phenome Archive. Graphs were constructed based on *IFITM1* and *IFITM3* nTPM, with cutoff established at 1 nTPM as the limit for reliable detection of genes in all organs and cell types. The consensus transcriptomic data of each gene were categorized according to the expression pattern as follows: (1) enriched, nTPM in a particular tissue/region/cell type at least 4 times any other tissue/region/cell type; (2) group enriched, nTPM in a group (of 2–5 tissues, brain regions, single cell types or cell lines, or 2–10 blood cell types) at least 4 times any other tissue/region/cell line/blood cell type/cell type; (3) enhanced, nTPM in 1 or several (1–5 tissues, brain regions or cell lines, or 1–10 immune cell types or single-cell types) at least 4 times the mean of other tissue/region/cell types; (4) low specificity, nTPM ≥1 in at least 1 tissue/region/cell type, but not elevated in any tissue/region/cell type; and (5) not detected, nTPM <1 in all tissue/region/cell types. HPA, Human Protein Atlas; IFITM, interferon-induced transmembrane; nTPM, normalized transcripts per million; RNA, ribonucleic acid; RNA-Seq, RNA sequencing.

SNPs affecting IFITM proteins have been linked to influenza susceptibility (Everitt and others 2012; Zhang and others 2013; Allen and others 2017; Kim and others 2020, 2021), but the effect of bulk *IFITM* expression on disease severity has not been addressed. Hence, we analyzed the relative mRNA expression of *IFITM1* and *IFITM3* in our cohort of patients with severe influenza A(H1N1), using HC and individuals with severe COVID-19 as reference. Since these determinations were performed in total PBMCs, we first retrieved bulk and single-cell RNAseq data from the Human Protein Atlas public dataset to identify the distribution of *IFITM1* and *IFITM3* expression in healthy conditions (Fig. 1).

Retrieved consensus normalized transcript per million (nTPM) values showed that *IFITM1* is enriched in the epididymis and enhanced in the ovary and spleen, whereas *IFITM3* is highly enhanced in the liver, adipose tissue, lung, kidney, cervix, and fallopian tube (Fig. 1A). Interestingly, analysis of mRNA expression in blood cells showed that *IFITM1* is more expressed than *IFITM3* in total PBMCs (Fig. 1B). Also, *IFITM1* was enriched in NK cells, T cells, and granulocytes (neutrophils and eosinophils), whereas *IFITM3* was expressed in neutrophils and monocytes.

**FIG. 2.**
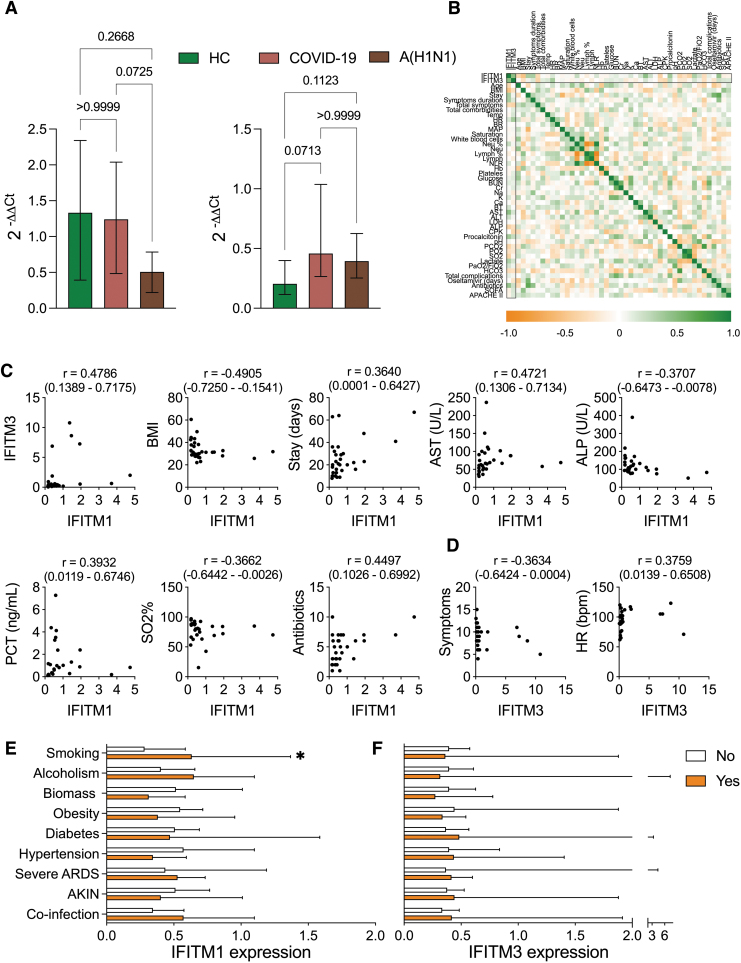
Distinctive patterns of *IFITM1* and *IFITM3* expression in patients with severe pandemic influenza A(H1N1). **(A)** Levels of *IFITM1* and *IFITM3* mRNA expression relative to *GAPDH* were determined in PBMCs from 8 HC, 31 patients with severe pandemic influenza A(H1N1), and 16 individuals with severe COVID-19. Kruskal–Wallis test/*post hoc* Dunn test. **(B)** Correlations between *IFITM1*/*IFITM3* and clinical variables were analyzed in patients with severe influenza using Spearman *r* coefficients. **(C)** Significant correlations of *IFITM1* with the following variables are individually showed: BMI, hospital stay, AST, ALP, PCT, SO_2_%, and number of antibiotics required during hospitalization. **(D)** Correlations of *IFITM3* with number of symptoms on admission and HR. **(E)** Expression values of *IFITM1* in participants with influenza were compared according to comorbidities, severe ARDS (PaO2/FiO2 < 100), and outcomes (AKIN, secondary co-infections). **(F)** Expression of *IFITM3* in patients with influenza grouped by comorbidities, disease severity, and outcomes. Unpaired Mann–Whitney *U* test. Graphs display medians and IQR. *P < 0.05. AKIN, acute kidney injury; ALP, alkaline phosphatase; ARDS, acute respiratory distress syndrome; AST, aspartate aminotransferase; BMI, body mass index; COVID-19, coronavirus disease 2019; *GAPDH*, glyceraldehyde 3-phosphate dehydrogenase; HC, healthy controls; HR, heart rate; IQRs, interquartile ranges; PBMCs, peripheral blood mononuclear cells; PCT, procalcitonin; SO_2_%, oxygen saturation.

Notably, as shown in Fig. 2A, in our cohorts we found that *IFITM1* was downregulated in PBMCs from patients with influenza, but not in the HC and COVID-19 groups, contrasting with the patterns observed in the data from the HPA dataset, although the differences were not statistically significant. Conversely, both pandemic influenza A(H1N1) and COVID-19 showed a modest, but significant upregulation of *IFITM3* compared to HC, which might reflect the higher circulation of monocytes and neutrophils during these infections. These data might indicate that viral infections change the normal expression profiles of these genes in the blood and suggest that different antiviral mechanisms operate during these infections. In fact, an ROC analysis showed that *IFITM1*, but not *IFITM3*, distinguishes severe pandemic influenza A(H1N1) from COVID-19 (Supplementary Fig. S1).

To evaluate the impact of *IFITM* expression during influenza, first, we looked for possible correlations with quantitative clinical features of affected patients (Fig. 2B). This analysis revealed a positive correlation between the levels of expression of *IFITM1* and *IFITM3* (Fig. 2C), even when their overall expression patterns looked contrasting when compared with HC and COVID-19 patients (Fig. 2A). Figure 2C also illustrates other correlations of *IFITM1* and clinical variables that resulted significant. Interestingly, the expression of *IFITM1* was inversely correlated with the body mass index of influenza patients, perhaps indicating that obesity affects this antiviral mechanism.

Diminished levels of *IFITM1* were also correlated with increased alkaline phosphatase concentrations, an enzyme that has been associated with pulmonary inflammation in patients with severe ARDS (Juschten and others 2020). In contrast*, IFITM1* showed positive correlations with days of hospital stay, levels of aspartate aminotransferase, PCT, and saturation of oxygen (SO_2_%) on admission, and the number of antibiotics required by patients later during their hospitalization (Fig. 2C). On the other hand, *IFITM3* was negatively associated with the number of symptoms reported by influenza patients and correlated with increased heart rate (HR) on admission (Fig. 2D).

Next, the *IFITM1* and *IFITM3* expression levels were compared between patients with pandemic influenza A(H1N1) grouped according to comorbidities, the severity of disease on admission, and in-hospital complications (Fig. 2E, F). This approach revealed that the expression of *IFITM1* was higher in patients who reported tobacco use, whereas a reduction in this gene was observed in those with obesity and exposure to biomass smoke, although the differences were not significant (Fig. 2E). In addition, *IFITM1* and *IFITM3* were similarly expressed in male and female patients (Supplementary Fig. S2). These results may indicate that demographic characteristics and previous clinical conditions do not influence levels of *IFITM* expression, and these later do not impact the subsequent incidence of relevant complications in patients with severe influenza. Hence, the gene expression profiles described in Fig. 2A might be explained by the effect of the viral infection.

**FIG. 3.**
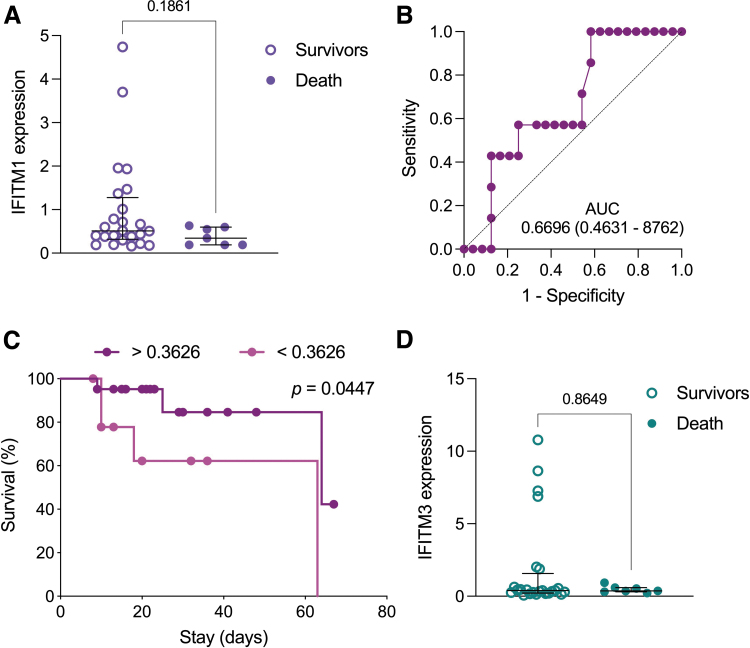
Prognostic value of *IFITM1* expression in severe pandemic influenza A(H1N1). **(A)** Relative *IFITM1* expression levels were compared between pandemic influenza A(H1N1) patients according to their clinical outcome (survival versus death). Unpaired Mann–Whitney *U* test. **(B)** AUC of *IFITM1* in survivor and deceased severe pandemic influenza A(H1N1) patients. **(C)** Survival curves were compared between pandemic influenza patients according to their *IFITM1* expression values using the Kaplan–Meier method and the log-rank test. The cutoff value for this analysis was estimated using the Youden index. **(D)** Relative *IFITM3* expression levels in pandemic influenza A(H1N1) patients by outcome. Unpaired Mann–Whitney *U* test. Graphs display medians and IQR. AUC, area under the ROC curve; ROC, receiver operating characteristic.

Regarding mortality, we also determined whether variations in *IFITM1* and *IFITM3* were associated with a higher risk of death. As observed in Fig. 3A, levels of *IFITM1* expression tended to be lower among patients with severe pandemic influenza A(H1N1) who succumbed to the infection compared to survivors. In fact, a survival curve comparison using a cutoff value determined by an AUC analysis showed that low levels of *IFITM1* expression, despite not being a good marker to differentiate individuals at mortality risk (Fig. 3B), are associated with lower survival (Fig. 3C). Meanwhile, *IFITM3* was expressed similarly between deceased and survivor influenza patients (Fig. 3D).

Longitudinal changes in the expression and activation of antiviral mechanisms might also determine disease severity and progression. For instance, previous studies have demonstrated that recovery in specific immune parameters predicts good outcomes in severe respiratory infections (Hernández-Cárdenas and others 2021). Hence, in a small subgroup of our influenza cohort from which we could obtain a second sample of PBMCs at intensive care unit (ICU) discharge, we analyzed longitudinal dynamic patterns of *IFITM1* and *IFITM3* expression and their relationship with the incidence of relevant in-hospital complications. Of note, initial *IFITM1* and *IFITM3* expression levels in influenza patients remained unchanged at the end of the follow-up period, and their dynamics were not associated with the risk of AKIN and secondary infection (Supplementary Fig. S3). Collectively, our results provide a first look at the impact of bulk mRNA *IFITM1* and *IFITM3* expression on clinical aspects of pandemic influenza A(H1N1) in severely ill patients.

### High-dose influenza A (H1N1) virus infection downregulates IFITM expression in mice

**FIG. 4.**
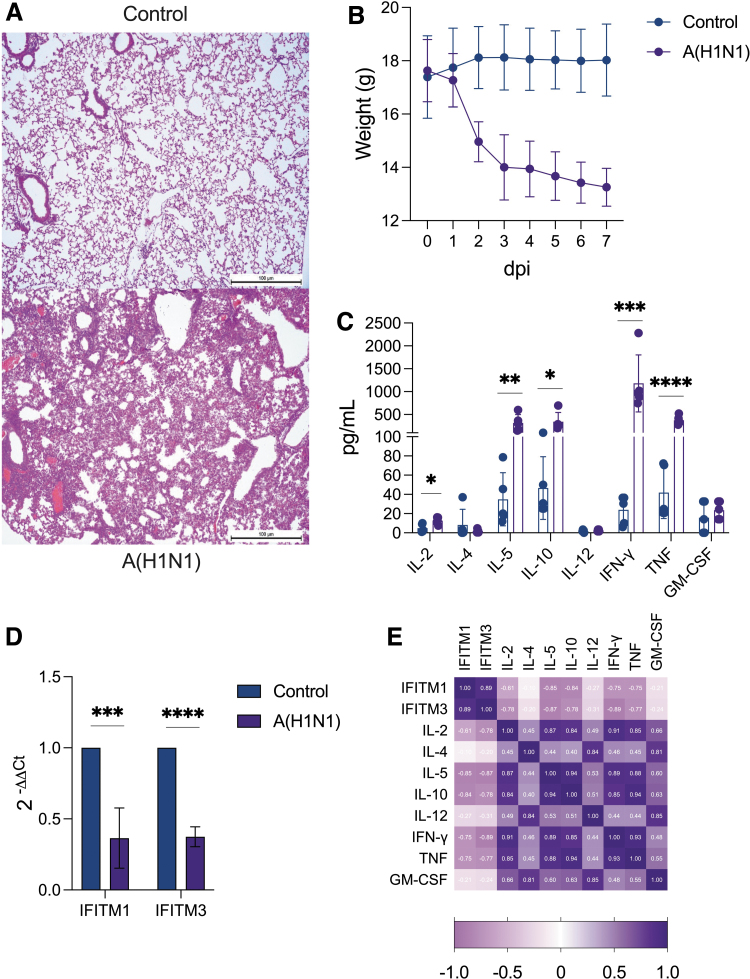
Severe pandemic influenza A(H1N1) virus infection downregulates *IFITM1* and *IFITM3* expression in mice. **(A)** Representative microphotographs showing severe pneumonia in H&E-stained lung sections from pandemic influenza A(H1N1)-infected C57BL/6 mice (*lower panel*) and uninfected controls (*upper panel*). **(B)** Weight of infected and control mice was monitored during the first 7 days postinfection. **(C)** At given time points, BAL samples were obtained from mice and the level of different cytokines determined by Luminex. Comparisons between groups were performed with the unpaired Mann–Whitney *U* test. **(D)** Expression of *IFITM1* and *IFITM3* in lung homogenates from infected and control mice was determined using the delta-delta *Ct* method relative to *GAPDH*. Unpaired Mann–Whitney *U* test. **(E)** Correlation matrix of *IFITM1/IFITM3* and lung cytokine levels in mice infected with pandemic influenza A(H1N1). Spearman correlation coefficient. Graphs display medians with IQR. ***P < 0.001, ****P < 0.0001. BAL, bronchoalveolar lavage; H&E, hematoxylin and eosin.

To confirm our previous observations, we turned to an animal model of severe pandemic influenza A(H1N1) established by infecting B6 mice intranasally with a high dose of a clinical viral isolate. As shown in Fig. 4, this model resembles histopathological aspects of severe pneumonia caused by influenza (Fig. 4A), characterized clinically by a rapid decline in animal health traduced by severe weight loss (Fig. 4B) accompanied by a cytokine storm (Fig. 4C). Remarkably, in this model, we found that severe pandemic influenza A(H1N1) downregulates both *IFITM1* and *IFITM3* expression in the lung (Fig. 4D). Furthermore, the expression of these genes showed significant inverse correlations with the levels of most cytokines measured in BAL specimens, except IL-4, IL-12, and GM-CSF (Fig. 4E).

**FIG. 5.**
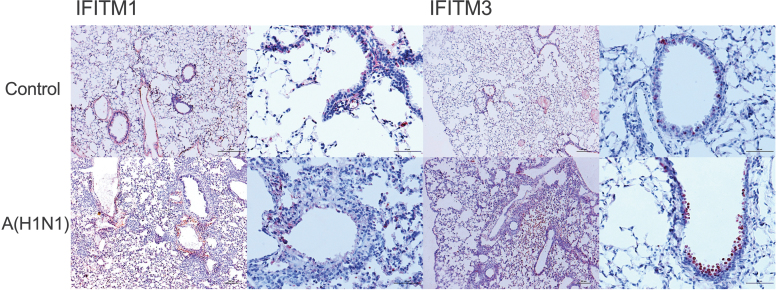
IFITM protein expression in the lungs of mice with pandemic influenza A(H1N1). Expression of IFITM1 and IFITM3 in lungs from mice infected with influenza A(H1N1) patients was assessed using specific antibodies by IHQ, × 100 (*left panels*) and × 400 (*right panels*). Staining intensity was determined according to the fraction of positive cells for each protein in each tissue (low, <25%; medium, 25%–75%; high >75%). IHQ, immunohistochemistry.

IHQ analysis confirmed the little IFITM1 and IFITM3 induction in mouse lungs infected with influenza, showing a restricted expression in some bronchial and alveolar epithelial cells, but not infiltrating leukocytes (Fig. 5). These findings coincide with human data regarding the low *IFITM1* expression found in both species with severe influenza, but the observations on *IFITM3* are contrasting. However, our human and mice analyses might indicate a direct influence of infection on the regulation of bulk *IFITM1* and *IFITM3* expression *in vivo*.

### IFITM1 and IFITM3 in severe COVID-19

Data from our comparative COVID-19 cohort might be relevant and deserve brief mentioning since very little is known so far about the role of IFITM proteins in antiviral defenses against SARS-CoV-2. In this context, only a few studies have found a correlation between the prevalence of SNPs affecting *IFITM3* and susceptibility to COVID-19 without influencing disease severity and mortality (Gómez and others 2021; Schönfelder and others 2021). Interestingly, as previously mentioned, in this study, we observed that *IFITM3*, but not *IFITM1*, is overregulated during severe COVID-19 (Fig. 2A). Further analysis showed that *IFITM1* was negatively correlated with HR, lymphocyte counts, and glucose levels in our COVID-19 cohort (Supplementary Fig. S4A). Interestingly, *IFITM1* and *IFITM3* expression levels were not correlated with most clinical characteristics and comorbidities of COVID-19 patients (Supplementary Fig. S4B, C**)** and did not influence in-hospital complications or mortality (Supplementary Fig. S4D, E).

## Discussion

The immune system has several antiviral mechanisms of protection, most of them highly dependent on IFN signaling. IFITM proteins are among those protective factors that limit host cell invasion by enveloped viruses. Nonetheless, viral pathogens have evolved clever ways to counteract immunity and ensure their access to the cell machinery. As such, viruses might evade IFNs and downstream IFN-dependent mechanisms by direct or indirect regulation. However, what is currently known about defensive molecules like IFITM proteins come from *in vitro* assays and observational studies in humans with viral infections, linking disease susceptibility to functional deficiencies genetically determined. The opposite side of the host-pathogen interaction where the virus plays a more active role downregulating IFITM functions has attracted less interest, despite its possible impact on morbidity and mortality.

In this study, we evaluated whether the bulk mRNA expression of *IFITM1* and *IFITM3* showed a particular pattern and dynamics during active severe pandemic influenza A(H1N1) virus infection in humans. Although several factors might influence *IFITM* expression beyond genetics, some of our findings might indirectly indicate that viruses could also regulate these mechanisms *in vivo*.

The main findings of this study were that *IFITM1* is downregulated in PBMCs from patients with influenza and is associated with lower survival in these patients. In contrast, *IFITM3* is overexpressed in both pandemic influenza A(H1N1) and COVID-19 and did not correlate with differences in survival. In line with these findings, in the experimental model of mice, we observed a limited expression of *IFITM1* in lungs from mice infected with high doses of pandemic influenza A(H1N1) strain, resulting in higher expression of immune mediators linked to the lung infiltrates of T cells and activated macrophages. Our human and mice analyses indicate a direct influence of influenza infection on the regulation of bulk *IFITM1* and *IFITM3* expression *in vivo*.

Importantly, IFITM2 and IFITM3 antiviral effects have been described against a broad range of RNA viruses (Jiang and others 2008, 2010; Brass and others 2009; Weidner and others 2010; Huang and others 2011; Lu and others 2011; Chan and others 2012). Experimental studies have demonstrated that these IFN transmembrane-related genes are relevant in the display of early protective IFN responses against HCV. A particularly important finding was that distribution of IFITM1 on cell membrane exerts a blocking effect of HCV cell entry. Interestingly, the responses mediated by IFITM2 and IFITM3 against HCV were observed in hepatocytes. In this regard, specific domains shared by IFITM proteins, such as S-palmitoylation, are essential for anti-HCV activity (Narayana and others 2015).

The importance of IFITM proteins in viral defense might also be dependent on the dynamics of viral entry and the stages of the disease at which these mechanisms are evaluated. For instance, Meischel and his colleagues reported that in experiments with 2 different strains of influenza virus with different mechanisms of cell entry, IFITM proteins are critical in the protection against these pathogens during early stages of infection, but their role to restrain the later stages of virus replication does not appear to be relevant (Meischel and others 2021). Indeed, the findings from these researchers indicate that IFITM1 acts only during early phases of influenza infection. This might partially explain why we observed distinct patterns of bulk *IFITM1* and *IFITM3* expression, since our patients were recruited several days after symptoms appeared.

Others have also reported that IFITM1 and IFITM3 have a different tissue-specific expression pattern during H9N2 infection in BALB/c mice (Yu and others 2015). Moreover, IFITM3 preferentially localizes in both early and late endosomes and lysosomal structures, so it can counteract the invading viruses more effectively and during more extended periods than IFITM1 (Lanz and others 2021). Furthermore, it has also been documented that besides preventing influenza A virus (IAV) fusion with the endosomal membrane, IFITM3 can also incorporate into IAV particles competing with viral hemagglutinin incorporation, therefore sensitizing the virus to antibody neutralization, and thus having an impact on the infection outcome (Lanz and others 2021).

Therefore, the expression and activity of IFITM3 might be more sustained compared with IFITM1 during the influenza A(H1N1) infection process. Despite this, seeing early stages of the infection at which IFITM proteins could participle is hard in human studies. However, our data in mice might provide insights into the early suppression of IFITM genes during acute severe influenza.

Regarding COVID-19, our findings in COVID-19 critically ill patients showed the expression of *IFITM3*, but not *IFITM1*. However, these molecules did not correlate with poor outcomes or mortality in these patients. These findings contrast with previous observations that IFITM proteins provide better protection to influenza viruses than SARS-CoV (Huang and others 2011). The variability of these observations might be influenced by genetic variations in IFITM genes. As such, genomic studies have revealed strong correlations of specific SNPs in the *IFITM3* gene and fatality in specific ethnic groups (Kim and Jeong 2020).

This finding is particularly important because it is possible that genetic background and transcriptional profiles of antiviral related genes might be determinant in the susceptibility to develop severe forms of the disease. Our group of patients was mainly from genetically admixed Mexican population, in which we have recently characterized specific genetic markers associated to susceptibility to inflammatory disorders and to differential transcriptional signatures that might be relevant in immune defense against pathogens (Torres-Garcia and others 2013; Zúñiga and others 2013; Olvera-García and others 2016; Hernández-Doño and others 2021).

As such, in previous reports, we have extensively analyzed the genetic structure of different Mexican Amerindian (ref Mayans), as well as Mexican admixed ethnic groups by using several genetic markers of ancestry, including SNPs, short tandem repeats, and major histocompatibility complex (MHC). As a result, we have found that specific populations in the south of Mexico such as Mayans have a reduced genetic diversity of MHC genes because of specific processes of genetic inbreeding (Barquera and others 2020).

In contrast, Mexican admixed population exhibits a greater contribution of Amerindian (41.3%); Caucasian (24.6%); a small contribution of African (6.7%); and Asian (3.0%) genes. Importantly, migration patterns of indigenous populations that inhabited around 100 km of Mexico City have shown an intense migration of indigenous individuals to Mexico City during the last 60 years, modifying the genetic diversity within the Metropolitan area of Mexico City (Zúñiga and others 2013).

Notably, it is well demonstrated that MHC diversity influences the responses against pathogenic microorganisms, and the effect of pathogen-driven immunity and population selection could be the result of co-evolutionary mechanisms at population level (Doherty and Zinkernagel 1975; Jeffery and Bangham 2000). More importantly, other studies have proposed that autoimmunity-related genes might be fixed in specific populations because of natural selective processes induced by endemic or emerging pathogens. In the group of severe COVID-19 patients who were recruited in this study, genetic admixture estimations using HLA class I and HLA class II MHC genes revealed 60% of Amerindian component, 30% of European component, and <10% of African genes (Zuñiga J, et al., unpublished data).

However, functional data and its correlation with genomic studies are crucial to explain whether the population genetic diversity of admixed populations could influence the clinical scenario in pathogen-enriched environments such as this pandemic of SARS-CoV-2. Overall, together with past studies (Gómez and others 2021; Schönfelder and others 2021), our findings might suggest a possible role for *IFITM3* against SARS-CoV-2 that warrants additional research.

Our study has some limitations to consider when interpreting the results. First, the small size of the studied population. This weakness was related with the lack of circulation of influenza virus in the last 2 years in Mexico as well as the restrictions to the student and researcher access to our institution due to the full conversion of our institute to the management of COVID-19 patients. Another limitation was the lack of analysis of the genetic variation of IFITM1 and IFITM3 genes and experimental assays of viral inhibition by these genes in specific conditions of infection with influenza A and SARS-CoV-2 virus. Finally, since we determined gene expression in total PBMCs from study participants, increases or decreases in gene expression observed in any leukocyte population are not apparent from our data. Hence, the changes observed may be due to differences in the circulation of various leukocyte populations in the blood.

In summary, this is the first study addressing bulk mRNA expression of *IFITM1* and *IFITM3* in peripheral leukocytes from patients with severe pandemic influenza A(H1N1) and their correlation with clinical outcomes. Our study provides evidence indicating that differential expression patterns of *IFITM* genes are characteristic features of pandemic influenza in severe disease. Furthermore, data from mice infected with a high dose of the virus suggest that the downregulation of *IFIMT1* and *IFITM3* might be involved in severe influenza pathogenesis. Finally, analyses in a comparative cohort also identify an induction of *IFITM3* expression and correlation of *IFITM1* with clinical variables in individuals with severe COVID-19, which deserve further investigation in more extensive studies. Collectively, our results add to the current literature supporting the role of IFITM proteins in protective immunity against respiratory viruses in humans.

## Supplementary Material

Supplemental data

Supplemental data

Supplemental data

Supplemental data

Supplemental data

Supplemental data
