# Supplementary material for: Differential Leukocyte Expression of IFITM1 and IFITM3 in Patients with Severe Pandemic Influenza A(H1N1) and COVID-19
Source: J Interferon Cytokine Res. 2022 Aug 18;42(8):430–43. doi: 10.1089/jir.2022.0036 (PMC9422779; doi:10.1089/jir.2022.0036)
Supplement: Supplemental data [file Suppl_TableS1.docx]

| **Table S1. Clinical characteristics of patients with severe COVID-19** | | |
| --- | --- | --- |
| **Characteristics** | **N = 16** | ***p* value *vs.* influenza** |
| Age (years), median (range) | 42.5 (24 – 73) | 0.0723 |
| Males | 10 (62.5) | 0.7422 |
| BMI | 29.1 (24.8 – 33.2) | 0.1511 |
| Comorbidities  Smoking  Alcoholism  Biomass exposure  Obesity  Diabetes  SAH  COPD | 4/12 (33.33)  3/12 (25)  0 (0)  5/12 (41.66)  1/12 (8.33)  0 (0)  0 (0) | 0.4995  0.7272  0.5629  0.3014  0.1393  0.0438  0.9999 |
| Symptoms at onset  Fever  Myalgia  Arthralgia  Headache  Dyspnea  Rhinorrhea  Sore throat  Thoracic pain  Dry cough  Productive cough  Fatigue  Diarrhea  Nausea  Vomit | 9/12 (75)  4/12 (33.33)  4/12 (33.33)  3/12 (25)  12/12 (100)  3/12 (25)  2/12 (16.66)  0 (0)  7/12 (58.33)  3/12 (25)  4/12 (33.33)  4/12 (33.33)  1/12 (8.33)  1/12 (8.33) | 0.1230  0.0318  0.0374  0.0995  0.5629  0.0995  0.4563  0.1627  0.4971  0.3065  0.3102  0.0419  0.9999  0.4850 |
| Duration of symptoms (days), median (range) | 6 (2 – 7) | 0.0826 |
| Triage vital signs  Body temperature (^o^C)  Respiratory rate (bpm)  Hearth rate (bpm)  MAP (mmHg)  SO_2_% | 37 (36.9 – 37.6)  24 (22 - 28)  99 (92 – 113)  88 (81.7 – 92.2)  88.4 (69.5 – 93) | 0.2234  0.8355  0.6160  0.4412  0.3581 |
| Stay in hospital (days) | 24 (15 – 38) | 0.7432 |
| Complications  Acute kidney injury  Secondary co-infection  Acute myocardial infarction  Deep vein thrombosis  Stroke | 5/12 (41.66)  6/12 (50)  0 (0)  0 (0)  0 (0) | 0.7449  0.2867  0.5478  0.9999  0.9999 |
| Medical treatment  Oseltamivir  Antibiotic therapy  No. of antibiotics/patient  Corticosteroids | 7/12 (58.33)  16 (100)  5 (3 – 7)  10/12 (83.33) | 0.0008  0.9999  0.5465  0.0004 |
| Intensive support  MV  Prone position  ECMO  RRT | 16 (100)  5/12 (41.66)  0 (0)  1/12 (8.33) | 0.9999  0.7360  0.5478  0.6520 |
| Mortality | 4/12 (33.33) | 0.4670 |
| Data are displayed as n (%) or median (IQR). N is the total number of patients with available data. BMI, body mass index; bpm, breaths/beats per minute; COPD, chronic obstructive pulmonary disease; ECMO, extracorporeal membrane oxygenation; IQR, interquartile range; ICU, intensive care unit; MAP, mean arterial pressure; MV, mechanical ventilation; PTB, pulmonary tuberculosis; RRT, renal replacement therapy; SAH, systemic arterial hypertension; SO2%, oxygen saturation. Comparisons with influenza patients were performed using the Fisher's exact test or Mann-Whitney U test, as appropriate. | | |
