# Supplementary material for: Differential Leukocyte Expression of IFITM1 and IFITM3 in Patients with Severe Pandemic Influenza A(H1N1) and COVID-19
Source: J Interferon Cytokine Res. 2022 Aug 18;42(8):430–43. doi: 10.1089/jir.2022.0036 (PMC9422779; doi:10.1089/jir.2022.0036)
Supplement: Supplemental data [file Suppl_TableS2.docx]

| **Table S2. Laboratory parameters of severe COVID-19 patients** | | |
| --- | --- | --- |
| **Parameters** | **N = 16** | ***p* value *vs.* influenza** |
| Blood counts  WBC (10^9^/L)  Neutrophils (10^9^/L)  Lymphocytes (10^9^/L)  NLR  Hgb (g/dL)  Platelets (10^9^/L) | 9.9 (7.3 - 14)  7.5 (5.9 – 12.7)  0.85 (0.6 – 1.5)  12 (7.3 – 15.4)  13.4 (12.2 – 14.6)  255 (205 – 372) | 0.0223  0.0640  0.2605  0.3175  0.0447  0.0012 |
| Metabolic parameters  Glucose (mg/dL)  Na (mmol/L)  K (mmol/L)  Ca (mg/dL) | 129.3 (91.6 – 162.7)  141 (137.8 – 142.8)  4.0 (3.7 – 4.1)  8.3 (7.8 – 8.6) | 0.1254  0.0005  0.0180  0.0916 |
| Renal function  Cr (mg/dL)  BUN (mg/dL) | 0.9 (0.6 – 1.1)  16.5 (11.1 – 22.8) | 0.0160  0.4024 |
| Liver function  Total bilirubin (mg/dL)  AST (U/L)  ALT (U/L) | 0.54 (0.39 – 0.58)  48 (24.2 – 81.6)  37.7 (29.2 – 60.2) | 0.0680  0.1093  0.4823 |
| Tissue injury markers  LDH (U/L)  ALP (U/L)  CPK (U/L)  PCT (ng/mL) | 360.5 (275.6 – 519.8)  77.1 (65.2 – 121.3)  85.5 (43.2 – 530.6)  0.1 (0.04 – 0.62) | 0.0016  0.0203  0.1259  0.0005 |
| Respiratory parameters  pH  PCO_2_ (mmHg)  PaO_2_ (mmHg)  Lactate (mmol/L)  HCO_3_^-^ (mEq/L)  PaO_2_/FiO_2_ (mmHg) | 7.43 (7.37 – 7.49)  32.1 (27.1 – 48.2)  51.2 (39.1 – 75.8)  1.4 (0.9 – 1.7)  25 (21 – 29.7)  139.4 (106.8 – 209.3) | 0.8988  0.2860  0.4415  0.9733  0.0089  0.1033 |
| Severity of illness  SOFA  APACHE II | 8 (4 – 10)  11 (6 – 16) | 0.4062  0.7530 |
| Data are displayed as n (%) or median (IQR). N is the total number of patients with available data. ALP, alkaline phosphatase; APACHE-II, Acute Physiology And Chronic Health Evaluation II; AST, aspartate aminotransferase; ALT, alanine aminotransferase; BUN, blood ureic nitrogen; CPK, creatine phosphokinase; Cr, creatinine; FiO_2_, fraction of inspired oxygen; HCO_3_, bicarbonate; Hgb, hemoglobin; IQR, interquartile range; K, potassium; LDH, lactate dehydrogenase; Na, sodium; NLR, neutrophil/lymphocyte ration; PaO_2_, partial pressure of oxygen in arterial blood; PCO_2_, partial pressure of carbon dioxide in the blood; PCT, procalcitonin; SOFA, Sequential Organ Failure Assessment; SO_2_%, oxygen saturation of blood; WBC, white blood cells. Comparisons with influenza patients were performed using the Fisher's exact test or Mann-Whitney U test, as appropriate. | | |
