# Supplementary material for: Differential Leukocyte Expression of IFITM1 and IFITM3 in Patients with Severe Pandemic Influenza A(H1N1) and COVID-19
Source: J Interferon Cytokine Res. 2022 Aug 18;42(8):430–43. doi: 10.1089/jir.2022.0036 (PMC9422779; doi:10.1089/jir.2022.0036)
Supplement: Supplemental data [file Suppl_FigS1.docx]

**
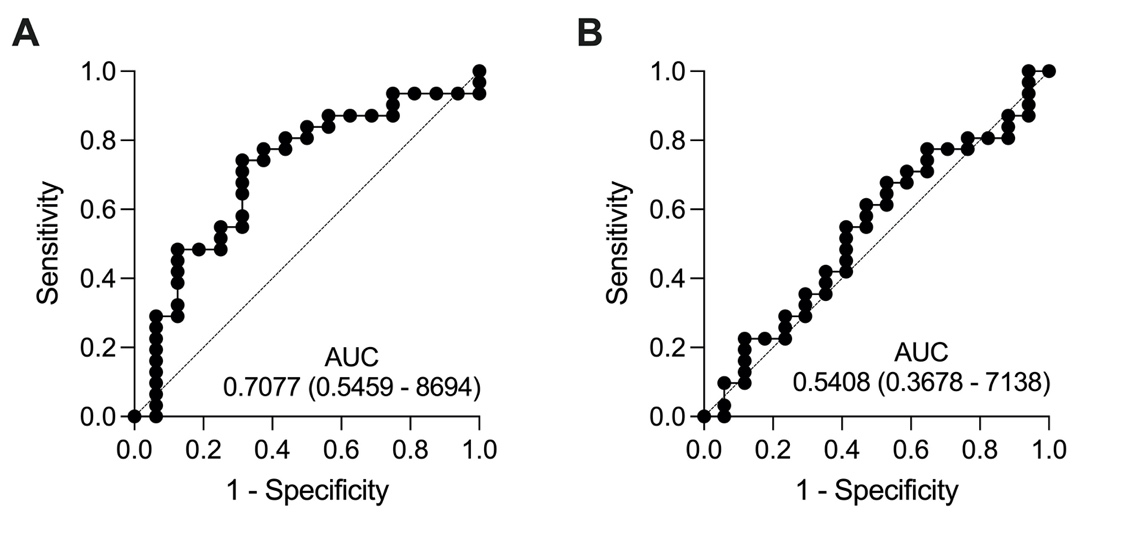
**

**Figure S1. *IFITM1* differentiates severe pandemic influenza (AH1N1) from COVID-19.** Receiver operating characteristic (ROC) curve of *IFITM1* **(A)** and IFITM3 **(B)** expression levels in patients with severe pandemic influenza A(H1N1) and COVID-19. The graphs display the area under de ROC curve (AUC) and 95% confidence intervals.
