# Supplementary material for: Differential Leukocyte Expression of IFITM1 and IFITM3 in Patients with Severe Pandemic Influenza A(H1N1) and COVID-19
Source: J Interferon Cytokine Res. 2022 Aug 18;42(8):430–43. doi: 10.1089/jir.2022.0036 (PMC9422779; doi:10.1089/jir.2022.0036)
Supplement: Supplemental data [file Suppl_FigS2.docx]

**
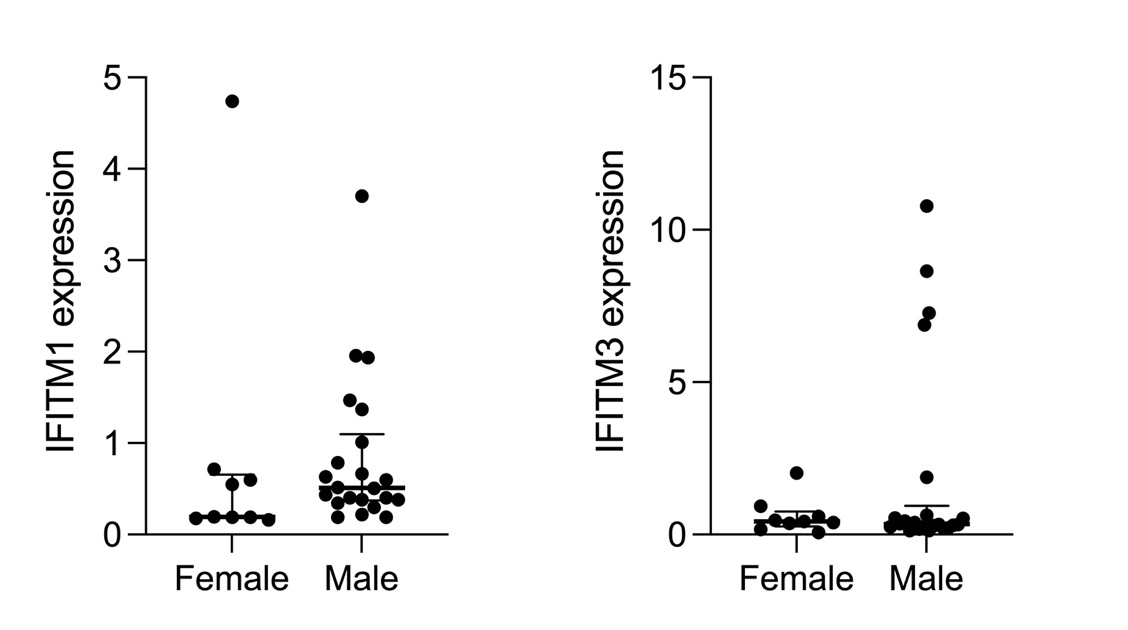
**

**Figure S2. *IFITM1* and *IFITM3* expression in patients with influenza according to gender.** Expression of *IFITM1* and *IFITM3* relative to *GAPDH* were evaluated using the double delta Ct method. Unpaired Mann-Whitney U test. Graphs display medians and interquartile range.
