# Supplementary material for: Differential Leukocyte Expression of IFITM1 and IFITM3 in Patients with Severe Pandemic Influenza A(H1N1) and COVID-19
Source: J Interferon Cytokine Res. 2022 Aug 18;42(8):430–43. doi: 10.1089/jir.2022.0036 (PMC9422779; doi:10.1089/jir.2022.0036)
Supplement: Supplemental data [file Suppl_FigS3.docx]

**
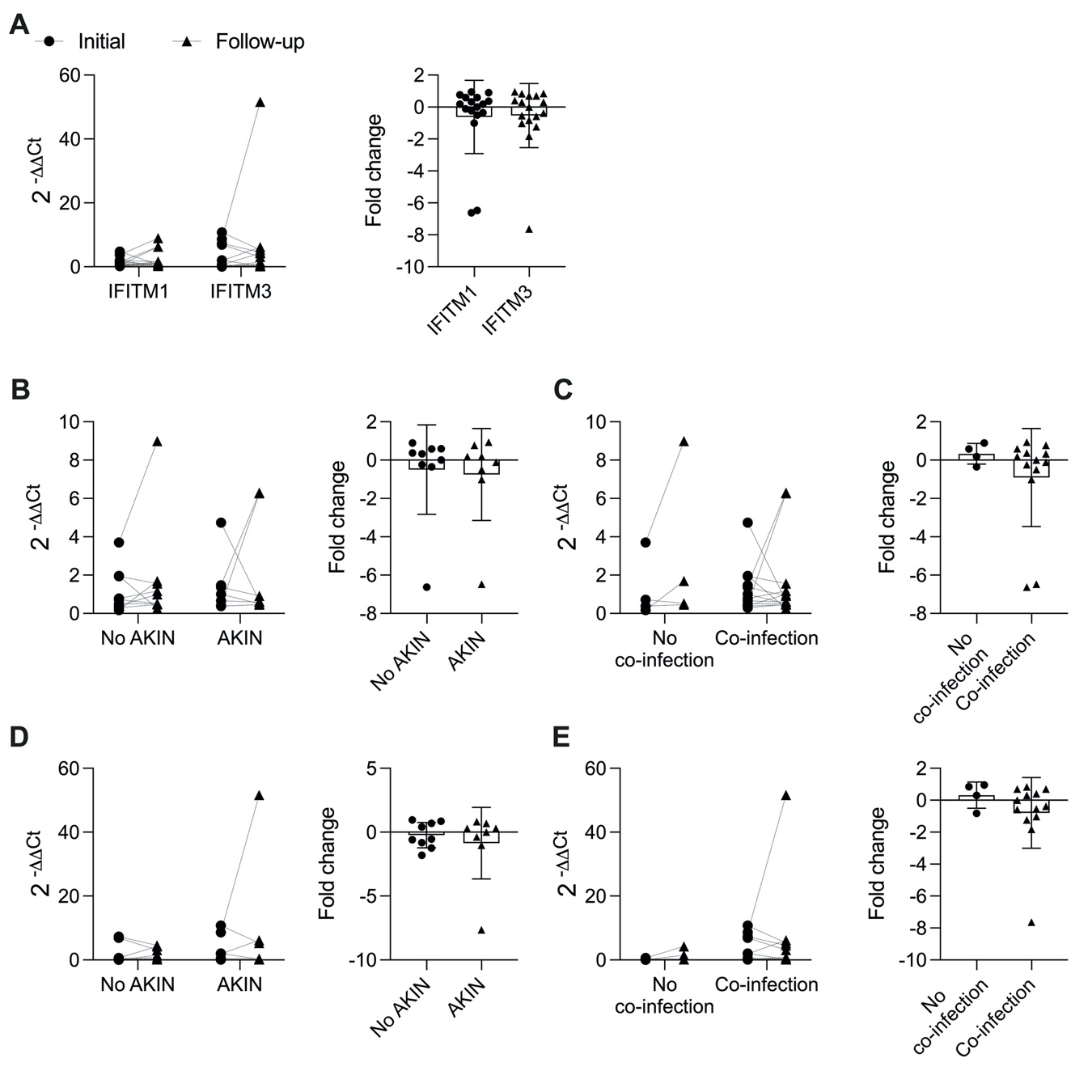
Figure S3. Longitudinal dynamics of *IFITM1* and *IFITM3* expression in patients with severe pandemic influenza A(H1N1).** *IFITM1* and *IFITM3* expression levels were determined in serial peripheral blood mononuclear cell (PBMC) samples of 17 pandemic influenza A(H1N1) patients taken at hospital admission (initial) and discharge from the intensive care unit (follow-up). Graphs display the longitudinal dynamics and magnitude of variations of both genes in terms of fold changes as follows: **A)** overall *IFITM1* and *IFITM3* expression; **B)** *IFITM1* in patients grouped according to the incidence of acute kidney injury (AKIN); **C)** *IFITM3* according to the incidence of secondary co-infections during hospitalization; **D)** *IFITM1* according to AKIN; **E)** *IFITM3* according to co-infections. Before-after comparisons were performed using the Wilcoxon signed-rank test. Fold changes between participants groups were compared with the unpaired Mann-Whitney U test. The graph displays medians with interquartile ranges.
