# Supplementary material for: Differential Leukocyte Expression of IFITM1 and IFITM3 in Patients with Severe Pandemic Influenza A(H1N1) and COVID-19
Source: J Interferon Cytokine Res. 2022 Aug 18;42(8):430–43. doi: 10.1089/jir.2022.0036 (PMC9422779; doi:10.1089/jir.2022.0036)
Supplement: Supplemental data [file Suppl_FigS4.docx]

**
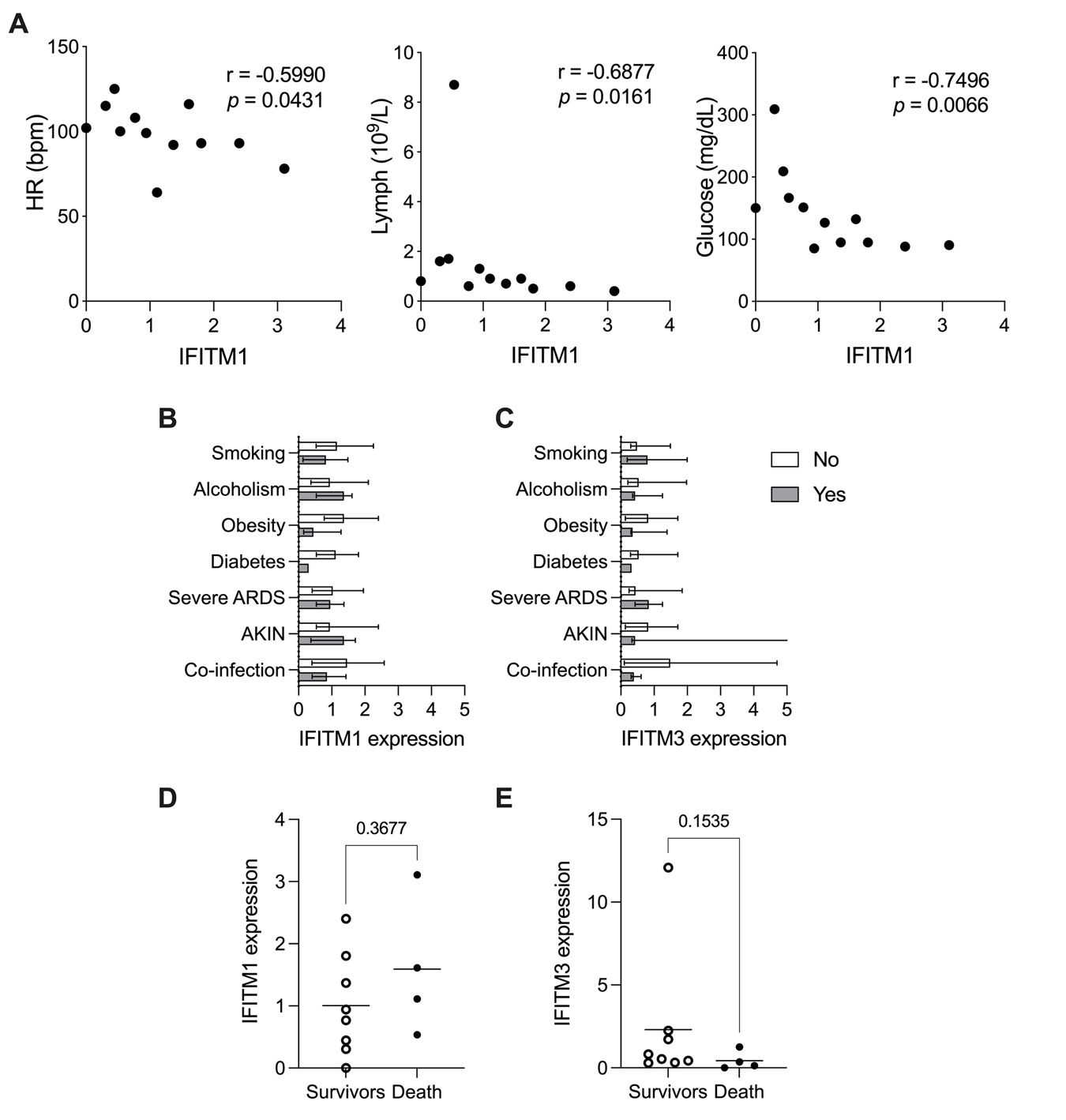
Figure S4. *IFITM1* and *IFITM3* expression in patients with severe COVID-19. A)** Significant correlations of *IFITM1* with heart rate (HR), lymphocyte counts, and blood glucose concentration in patients with severe COVID-19 were determined using the Spearman correlation coefficient. **B)** Expression values of *IFITM1* in participants with COVID-19 were compared according to comorbidities, severe acute respiratory distress syndrome (ARDS; PaO2/FiO2 <100), and outcomes (acute kidney injury (AKIN), secondary co-infections). **C)** Expression of *IFITM3* in patients with COVID-19 grouped by comorbidities, disease severity, and outcomes. **D)** Relative *IFITM1* expression levels were compared between COVID-19 patients according to their clinical outcome (survival vs. death). **E)** IFITM3 expression according to outcome. Unpaired Mann-Whitney U test. Graphs display medians and interquartile ranges.
